# Microbial co-inoculation and extracellular vesicles: new frontiers for soybean productivity

**DOI:** 10.1007/s10482-026-02337-3

**Published:** 2026-05-20

**Authors:** Fernando Sintra Fulaneti, Edgar Salis Brasil-Neto, Vítor Sauzem Rumpel, Laís de Paula Ribeiro, Lucas Nascimento Brum, Lucas Pedro Cipriani, Thomas Newton Martin

**Affiliations:** Department of Crop Science, Universidade Federal de Santa Maria, Santa Maria, Brazil

**Keywords:** *Glycine max* L., Microorganisms, Inoculants

## Abstract

Over the past decades, the intensive use of chemical fertilizers in agriculture has shown low efficiency while causing serious environmental issues and leading to soil nutrient imbalances. These challenges are compounded by climate change, increasing incidence of diseases and pests, and soil acidification, factors that jeopardize agricultural productivity and, consequently, threaten global food security. Soybean (*Glycine max* L.) is one of the world’s most important crops, serving as a key source of protein and oil for both human consumption and animal feed. Its global relevance continues to grow with rising demand for food, biofuels, and industrial applications, with Brazil, the United States, and Argentina leading production. Beyond its economic value, soybean contributes to agricultural sustainability through symbiotic nitrogen fixation, reducing the need for synthetic fertilizers. However, maintaining high yields under changing environmental conditions requires innovative management strategies. In this context, one promising strategy to mitigate these problems is the use of plant growth-promoting bacteria (PGPB), which contribute to more sustainable crop yield. Although numerous studies are underway regarding the potential of PGPB, further research is still necessary due to the limited understanding of their mechanisms of action and the vast range of benefits they may offer. Currently, there is a wide variety of inoculants based on different bacterial species, which play a key role in stimulating plant growth and reducing reliance on agrochemicals. Among emerging technologies, noteworthy examples include molecular inoculants (still not widely adopted commercially), bacterial and fungal consortia formulated into a single product, and inoculants containing genetically edited microorganisms—all of which have shown great promise in enhancing the performance of beneficial microbial species. The selection and genetic editing of rhizosphere-associated PGPB—an essential component of the plant microbiome—are viable alternatives for promoting more sustainable agriculture. Thus, this review examines the main inoculant technologies aimed at obtaining efficient microorganisms capable of improving rhizosphere conditions and microbial community dynamics, representing a strategic opportunity for developing solutions that enhance soybean sustainability.

**Graphical abstract:**

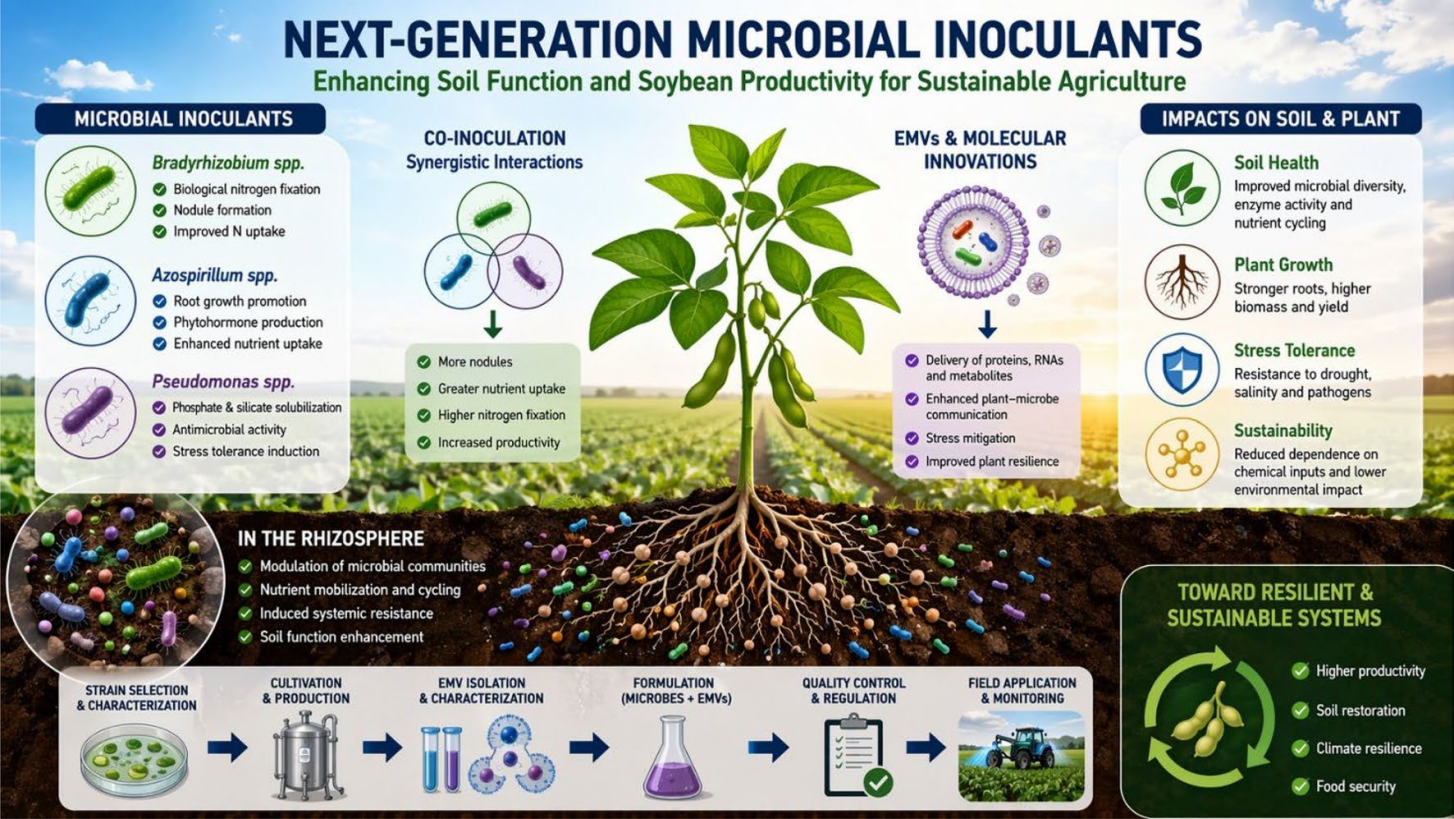

Rhizosphere processes mediated by microbial co-inoculation and extracellular vesicles: impacts on soil function and soybean Sustainability.

## Introduction

Soybean [*Glycine max* (L.) Merr.] has become one of the most economically important commodities in recent decades, both for Brazil and globally. Currently, Brazil leads the world in soybean production, accounting for approximately 40% of global output (United States Department of Agriculture [USDA] 2026). Soybeans are known for their high protein content and have a wide range of applications, notably in human nutrition, animal feed, and the chemical industry.

Among the viable strategies to address the challenges of modern agriculture, the use of plant growth-promoting bacteria (PGPB) stands out. These challenges include climate change and the increasing frequency of extreme weather events, soil degradation such as loss of organic matter, compaction and acidification, low efficiency in fertilizer use, and rising pressure from pests, diseases, and weeds. In addition, there is a growing need to sustainably increase productivity to meet global demand. PGPB can mitigate the adverse effects of salt and water stress, assist in controlling phytopathogens, enhance phosphorus solubilization, and regulate plant growth and development, thereby promoting more sustainable agriculture on a global scale (Santoyo et al. 2016).

Despite the significant growth in soybean production in many regions between 1975 and 2010, a 15% average decline in yields has been observed in recent decades, mainly due to unfavorable climate conditions (Gerber et al. 2024). This scenario presents additional challenges, especially in light of intensifying climate change and the urgent need to increase food production to meet the demands of a projected global population of 10 billion by 2050 (Un 2020). Climate projections for the coming decades suggest significant changes, including rising global temperatures, irregular rainfall patterns, expanding areas affected by soil salinization, and increased plant disease incidence, among other adverse conditions (El-esawi et al. 2018; Yasmin et al. 2020).

In response to this context, several studies have explored the potential of different bacterial genera and species associated with soybean cultivation (Barbosa et al. 2021). However, it is essential to conduct further research to validate the effectiveness of each bacterial species under specific growing conditions and environments. Although progress is still needed, some bacterial species have already shown positive effects on soybean crops. The genus *Azospirillum* spp., for instance, stands out for its ability to synthesize phytohormones that stimulate root and shoot growth, leading to increased productivity (Cassán and Dias-Zorita 2016). Similarly, species of *Pseudomonas* spp. have demonstrated important capabilities, such as pathogen control, induction of systemic resistance, phosphorus (P) and potassium (K) solubilization, and the production of hormones, antibiotics, and secondary metabolites, especially under stress conditions (Mehmood et al. 2023).

Bacteria of the genus *Bradyrhizobium* spp. are widely recognized for their efficiency in nodule formation and symbiotic nitrogen fixation (SNF). Their tolerance to saline stress represents an additional advantage and may contribute to enhanced plant growth (Meng et al. 2016). The co-inoculation of soybean with *Azospirillum* spp. and *Bradyrhizobium* spp. has been increasingly adopted, as it enhances the number and mass of nodules, as well as root and shoot biomass (Silva et al. 2019).

Currently, most research focuses on evaluating soybean responses to biotic and abiotic stresses, including water stress, salinity, and pathogen infection, in addition to investigating variables such as root morphology, nodule number and mass, germination rate, and plant biomass (Cerezini et al. 2016; Queiroz Rego et al. 2018). However, many of these studies lack an integrated agronomic approach that would allow for a more precise identification of the conditions under which PGPB are most effective. Therefore, it is essential to target the use of PGPB toward specific stress situations, such as drought, pathogen attacks, or saline environments, in order to maximize crop yield. In this context, several technologies have been adopted in a complementary manner to mitigate stresses and enhance productive efficiency, including co-inoculation and the use of microbial consortia, the development of advanced inoculant formulations, and the on-farm production of microorganisms. Non-microbial biostimulants also stand out, along with the application of precision agriculture tools focused on the use of bioinputs and the use of digital platforms combined with agronomic modeling to support decision-making. In parallel, advances in gene editing and in the selection of more efficient microorganisms, as well as the adoption of integrated biological management systems and the use of nanotechnology in agriculture, expand the potential of these approaches. In this scenario of technological intensification and integration of practices, the use of extracellular membrane vesicles (EMVs) emerges as a promising innovation within a broader set of emerging solutions. Thus, the objective of this research is to understand the current state of knowledge regarding the use of microorganisms in agriculture in order to support the interpretation of ongoing research results and guide the planning of future investigations.

## Soybean production and challenges in Brazil

### Strategic relevance of soybean in the global and Brazilian context

Soybean is one of the most strategic crops in the global agri-food system, with worldwide production exceeding 426.82 million tons. In this context, Brazil has consolidated its position as the world’s leading producer, with an estimated output of 175 million tons in the 2024/2025 season, cultivated across more than 48 million hectares (USDA 2026). The country’s strong integration into international markets is evidenced by the fact that 62.59% of its production is exported, with China as the primary destination (Companhia Nacional de Abastecimento [Conab] 2025a). Beyond its economic importance, soybean exhibits high functional complexity and versatility. Its grains are rich in bioactive compounds, including lipids, fatty acids, polyphenols, antioxidants, and soluble fibers (Medic et al. 2014; Islam et al. 2019), supporting its widespread use in food, industrial, and energy sectors. Soybean derivatives are utilized in the production of oils, surfactants, polymers, paints, lubricants, and fibers (De Pretto et al. 2018; Brentin 2014). Additionally, soybean biomass presents potential for bioenergy conversion due to its hemicellulose and cellulose content, although its efficiency remains lower than that of crops such as sugarcane and corn (Davis et al. 2013; Islam et al. 2019). This multifunctionality positions soybean at the intersection of food security, energy transition, and the bioeconomy.

### Production intensification and integration into the energy matrix

The recent expansion of soybean cultivation in Brazil is closely associated with production intensification and increasing integration into the national energy matrix. The growth of biodiesel production, largely derived from soybean oil, contributes significantly to the mitigation of greenhouse gas emissions (Da Silva César et al. 2019; De Oliveira and Coelho 2017). This process is reinforced by public policies that progressively increase the proportion of biodiesel in commercial diesel, consolidating soybean as a key component of the energy transition. Moreover, soybean has a unique agronomic advantage due to its symbiotic association with nitrogen-fixing bacteria, which reduces the need for synthetic nitrogen fertilizers. This characteristic not only lowers production costs but also mitigates environmental impacts associated with fertilizer manufacturing and application (Da Silva César et al. 2019), reinforcing its role in more sustainable agricultural systems.

### Structural soil constraints and agronomic implications

Despite its high productive potential, soybean expansion in Brazil predominantly occurs in highly weathered tropical soils characterized by significant physicochemical constraints. Approximately 67% of the national territory presents soil pH values below 5.5 (Crespo-Mendes et al. 2019), a condition that limits nutrient availability and increases aluminum toxicity, directly affecting root development and crop productivity (Lin et al. 2012). Agricultural frontier regions such as MATOPIBA are dominated by sandy soils with low water retention capacity and reduced natural fertility (Dos Santos et al. 2024). These soils typically exhibit low levels of essential nutrients, including available phosphorus (< 1.2 ppm), potassium (69–128 ppm), and organic matter (Prado et al. 2012; Pavinato et al. 2020; Neri et al. 2013), making production systems highly dependent on external inputs. This situation is further intensified by the rapid expansion of cultivated areas in Brazil, which has led the country to become one of the world’s leading importers of fertilizers, with imports reaching 44.3 million tons in 2024 (Menezes et al. 2024; Conab 2025b). In this context, microorganisms play a fundamental role by acting synergistically to promote plant growth, enhancing nutrient and water uptake. However, soil acidity compromises these biological interactions, negatively affecting the performance of bioinputs, particularly those based on plant growth-promoting bacteria (PGPB), and impairing nodulation by *Bradyrhizobium japonicum* (Lin et al. 2012; Msimbira and Smith 2020).

### Climate change and the reconfiguration of phytosanitary systems

Climate change introduces an additional layer of complexity to agricultural systems by directly influencing the dynamics of pathogens, vectors, and soil microorganisms. Variations in temperature and precipitation patterns may accelerate disease emergence, alter ecological interactions, and reduce the functional diversity of beneficial microbiota (Priya et al. 2024; Shahzad et al. 2021). In Brazil, the combination of tropical climate conditions, high humidity, and continuous cropping favors the persistence and accumulation of pathogen inoculum (Lin et al. 2022). In this context, practices such as crop rotation and the use of resistant cultivars become essential components of phytosanitary management. Crop rotation, in particular, disrupts the life cycles of pathogens, nematodes, and insect pests by introducing non-host species, thereby reducing biotic pressure and enhancing system stability (Ratnadass et al. 2012; Heinrichs and Muniappan 2019).

### Pest and disease complex and impacts on productivity

Soybean productivity is strongly influenced by interactions with a diverse complex of pests and diseases. The nematode *Heterodera glycines* stands out as a major constraint, with wide geographic distribution and the potential to cause yield losses exceeding 30% (do Nascimento et al. 2016; Lima et al. 2017; Wendimu 2022). Among soilborne pathogens, *Fusarium oxysporum* and *Phytophthora sojae* are particularly aggressive, causing seedling damping-off and root rot, with yield losses that may reach up to 100% under favorable conditions (Arias and Leandro 2013; Sugimoto et al. 2012). Bacterial diseases, such as those caused by *Xanthomonas axonopodis* pv. glycines, also pose significant threats, with productivity reductions of up to 57.61% (Savitri et al. 2023). In addition, insect pests such as *Helicoverpa armigera* significantly impact yield, with damage varying according to the crop’s reproductive stage (Gopalakrishnan et al. 2016; Stacke et al. 2018). In this context, various microorganisms are increasingly used not only for biological pest control but also for the suppression of pathogenic fungi, contributing to more sustainable and efficient agricultural management. This scenario highlights the need for integrated management strategies grounded in both ecological understanding and technological innovation.

### Biological control and sustainable intensification via the microbiome

The growing demand for sustainable agricultural practices has driven the adoption of microorganisms as biological control agents and plant growth promoters. Species within the genus *Bacillus* have demonstrated broad efficacy in managing nematodes and soilborne pathogens, including the suppression of *Fusarium oxysporum* and *Phytophthora sojae* by *Bacillus siamensis* HT1 (He et al. 2023; Zhou et al. 2024). Microorganisms such as *Pseudomonas chlororaphis* IRHB3 play a role in controlling phytopathogenic fungi (Wei et al. 2023), while *Bacillus amyloliquefaciens* KPS46 can induce systemic resistance in soybean plants against bacterial pathogens (Buensanteai et al. 2009). For insect control, *Streptomyces* sp. has shown effectiveness against *Helicoverpa armigera* (Gopalakrishnan et al. 2016). These advances reflect a paradigm shift in which the agricultural microbiome is increasingly recognized as a central component of productivity and resilience in cropping systems.

## Soil fertility management and optimization of biological processes

Overcoming soil limitations requires integrated management strategies combining chemical, physical, and biological approaches. Liming is a fundamental practice for correcting soil acidity, enhancing nutrient availability, and promoting microbial activity (Alves et al. 2023). Maintaining soil pH between 6.0 and 6.5 is critical to maximize nodulation by *Bradyrhizobium japonicum* and the efficiency of biological nitrogen fixation (Comissão de Química e Fertilidade do Solo–RS/SC [CQFS] 2016; Lin et al. 2012; Hungria andMendes 2015; Stecca et al. 2018). Additionally, the selection of strains adapted to Cerrado conditions has improved the performance of inoculants (Bender et al. 2022). Furthermore, microorganisms such as *Pseudomonas* spp. play key roles in phosphorus solubilization and in enhancing plant immunity, contributing to improved resource-use efficiency and agronomic performance (Shahid et al. 2018; Dubey et al. 2021).

### Integrated strategies and future perspectives

Soybean remains a cornerstone of global agricultural systems, playing a strategic role in food security, energy production, and the bioeconomy. However, challenges associated with soil constraints, climate change, and biotic stresses demand a reconfiguration of production systems. In this context, integrating soil management practices, bioinputs, and plant–microorganism interactions represents a promising pathway for sustainable intensification. Advances in microbiome research and emerging technologies are expected to redefine agricultural paradigms, enabling the development of more resilient, efficient, and environmentally sustainable cropping systems.

## Plant-microorganism interaction in agriculture

Plant growth-promoting bacteria (PGPB) are soil microorganisms that predominantly inhabit the rhizosphere but are also capable of colonizing the root interior (endosphere) and aerial plant surfaces such as the phyllosphere. These microorganisms are directly or indirectly involved in promoting plant growth, mainly through the production and secretion of various metabolites widely used in agriculture (Poria et al. 2022). The rhizosphere constitutes a highly dynamic ecological niche and represents the primary zone of interaction between plants and microorganisms. The rhizosphere is a soil region surrounding plant roots, typically ranging from 0.5 to 4 mm in thickness (Kuzyakov and Razavi 2019). In this zone, plants release a variety of compounds such as sugars, phenolics, lipids, proteins, and nucleic acids, which shape the composition and dynamics of the associated microbial community and promote plant growth (Sugiyama 2019). These root exudates act as both chemical signals and energy sources, regulating microbial recruitment and activity. These bacteria are associated with the rhizosphere, a soil zone characterized by intense interactions between plants and microorganisms (Ling et al. 2022). PGPB assist in the uptake of essential resources such as nitrogen (N), phosphorus (P), and other minerals (Roriz et al. 2020).

Moreover, they influence hormone levels, mitigate pathogen effects, and promote overall plant growth and development (Timofeeva et al. 2024). The rhizospheric microbiome is typically more specialized and functionally diverse than that of bulk soil (Pantigoso et al. 2022; Ayilara et al. 2023), accumulating microorganisms that enhance plant performance as an adaptive strategy (Li et al. 2021). Its composition is strongly influenced by agricultural practices, environmental conditions, and plant-related factors such as genotype, phenological stage, and crop rotation systems (Longley et al. 2020; Ayilara et al. 2023).

Plant growth-promoting bacteria (PGPB) enhance plant development through a combination of direct and indirect mechanisms that operate at physiological, biochemical, and molecular levels. Direct mechanisms are primarily associated with improved nutrient acquisition, including biological nitrogen fixation, phosphate solubilization via the release of organic acids, and iron mobilization through siderophore production, all of which increase nutrient bioavailability and uptake efficiency (Olanrewaju et al. 2017; Abdelaal et al. 2021). In addition, many PGPB synthesize phytohormones such as auxins, cytokinins, and gibberellins, which modify root architecture, increasing root surface area and enhancing water and nutrient absorption capacity (Etesami 2025). Another key mechanism involves hormonal modulation under stress conditions, particularly through the production of ACC deaminase, which reduces ethylene levels and alleviates stress-induced growth inhibition, thereby promoting root elongation and plant vigor (Pérez-Montaño et al. 2025). In addition to supporting primary metabolic processes such as plant growth and nutrient uptake, illustrated by root growth stimulation by *Azospirillum* spp. and phosphorus solubilization by *Pseudomonas* spp., plant growth-promoting bacteria (PGPB) also play a significant role in secondary plant metabolism (Koza et al. 2022). This occurs, among other mechanisms, through the production and synthesis of plant hormones such as auxins, abscisic acid, and gibberellins, which are key regulators of secondary metabolism (Koza et al. 2022; Lv et al. 2024).

Among the major secondary metabolites produced are benzopyrones, quinones, flavonoids, tetralones, xanthones, terpenoids, phenolic compounds, steroids, and tannins (Narayanan and Glick 2022). Indirect mechanisms are mainly related to plant protection against biotic stresses, involving the production of antibiotics, lytic enzymes such as chitinases, and competition for ecological niches and resources in the rhizosphere (Olanrewaju et al. 2017). Additionally, PGPB can activate induced systemic resistance (ISR), a plant-wide defense response mediated by signaling pathways involving jasmonic acid and ethylene, enhancing resistance to a broad spectrum of pathogens (Etesami 2025). These mechanisms operate in an integrated manner, and their synergistic effects result in increased plant resilience, productivity, and resource-use efficiency, particularly under adverse environmental conditions (Pérez-Montaño et al. 2025).

Among the most widely used PGPB in agriculture, species belonging to the genera *Azospirillum* and *Pseudomonas* stand out due to their effectiveness and relatively low cost (Dos Reis et al. 2024). These microorganisms act primarily through phytohormone production, nutrient solubilization, and modulation of plant stress responses. In contrast, *Bradyrhizobium* spp. exhibit a highly specialized symbiotic mechanism and should be considered separately. These bacteria perform symbiotic nitrogen fixation (SNF) in soybeans by establishing a specific symbiotic relationship that results in the formation of root nodules. Within these nodules, differentiated bacterial cells known as bacteroids convert atmospheric nitrogen (N₂) into ammonia, a form assimilable by plants (Nakei et al. 2022). Despite their relatively slow growth, they play essential ecological roles, including the induction of nodulation and participation in processes such as symbiotic denitrification (Jones et al. 2016). The symbiosis between soybean and *Bradyrhizobium japonicum* involves a highly regulated molecular dialogue mediated by the exchange of chemical signals, including flavonoids exuded by plant roots and Nod factors synthesized by the bacteria (Bogino et al. 2015). This interaction triggers a cascade of events leading to nodule organogenesis. The process begins with the activation of bacterial nodulation genes (nod, nol, noe), followed by the production of lipo-chitooligosaccharides that induce root hair curling and cortical cell division, ultimately resulting in the formation of functional nodules where BNF occurs (Rodríguez-Navarro et al. 2011). Beyond its agronomic importance, *Bradyrhizobium japonicum* may also contribute to mitigating greenhouse gas emissions, as certain strains possess the nosZ gene, which encodes nitrous oxide reductase capable of reducing N_2_O to N_2_ during denitrification (Klimasmith and Kent 2022).

The successful application of these microorganisms in agriculture depends on efficient inoculation strategies and formulation technologies. Inoculants can be applied either to seeds or directly into planting furrows, with the latter often providing better moisture conditions and improved bacterial survival (Martin et al. 2023). Upon radicle emergence, plants release chemical signals such as flavonoids and betalains that attract beneficial microorganisms and initiate rhizosphere interactions. Continuous use of inoculants, particularly those based on *Bradyrhizobium*, can lead to the establishment of naturalized populations that integrate into the native soil microbiota (Hungria et al. 2020). However, the widespread use of chemically treated seeds poses challenges to microbial survival. To ensure inoculant effectiveness, the use of protectants has emerged as a practical strategy to minimize the negative effects of chemical seed treatments (Martin et al. 2023; Fipke et al. 2020). Among these, osmoprotectants are particularly effective, reducing bacterial mortality and increasing grain yield by up to 10.8% (Stecca et al. 2018), while polymer-based encapsulation technologies enable controlled microbial release and improved survival during storage and field application (Vassilev et al. 2020). Emerging approaches, such as the use of membrane vesicles, represent a promising frontier for enhancing microbial performance, with potential benefits for root development, nodulation, biological nitrogen fixation efficiency, and plant immunity.

Root exudates play a central role in shaping microbial community structure and function, influencing both microbial recruitment and symbiotic efficiency (Sugiyama 2019; Liu et al. 2021). Inoculation with PGPB can significantly restructure the rhizosphere microbiome, increasing system resilience and enhancing the functional performance of biological products (Ray et al. 2020). Despite advances, it remains unclear to what extent the native soybean microbiome directly influences rhizobial nodulation (Liu et al. 2019), highlighting the need for further research on plant–microbiome interactions. Among the microorganisms associated with soybean, *Bradyrhizobium* spp. stands out due to its well-established symbiotic efficiency and its central role in biological nitrogen fixation, making it a key component of sustainable agricultural systems.

## The prominence of *Bradyrhizobium japonicum* in sustainable agriculture

The *Bradyrhizobium* genus comprises nitrogen-fixing bacteria (NFB) capable of forming nodules on the roots of leguminous plants (Ormeño-Orrillo and Martínez-Romero 2019). In soybean production systems, this symbiosis represents the primary biological pathway for nitrogen acquisition, making *Bradyrhizobium* a central component of crop productivity and sustainability. The symbiosis between these bacteria and the host plant occurs through the formation of root nodules, where the plant houses and nourishes the bacteria by supplying nutrients and carbohydrates. In return, the bacteria capture atmospheric nitrogen and, through the enzyme nitrogenase, reduce it to ammonia in a reaction that consumes 16 ATPs (Lima et al. 2024), which is later transformed into nitrogen compounds assimilable by soybean plants (Ochieno et al. 2021). The bacteria infect the root system through molecular signaling, leading to nodule formation and subsequent symbiosis, in which the bacteria provide essential nitrogen for soybean development in exchange for photosynthates produced by the plant (Maróti and Kondorosi 2014). For every 1000 kg of soybean grains produced, 68.5 kg of nitrogen is required during the crop cycle (Martin et al. 2023; Salvagiotti et al. 2021), and BNF can supply up to 84% of this nitrogen (Martin et al. 2023; Gelfand and Robertson 2015).

At the molecular level, the establishment of this symbiosis is a highly regulated process that directly determines the efficiency of symbiotic nitrogen fixation. During germination, seeds release a variety of molecules, some of which attract rhizobia, others stimulate bacterial growth near the root, and others activate bacterial genes involved in nodulation (Passaglia 2017). These genes code for Nod factors, which initiate the root infection process (Zhang et al. 2021). Infection begins with the formation of an entry point, promoting cell division in the root cortex (Sarao et al. 2024; Fan et al. 2022), ultimately leading to the development of a nodule primordium (Fan et al. 2022). Once the infection thread reaches the primordium cells, the bacteria are released into the host cytoplasm, forming a specialized organelle called the symbiosome, which houses the bacteroids, the differentiated form in which they perform nitrogen fixation (Coba de la Peña et al. 2018; Bomfim et al. 2021).

After the nodule forms, various enzymes are synthesized, notably nitrogenase, responsible for molecular nitrogen fixation, along with other proteins essential for bacterial metabolism (Nakei et al 2022). In infected nodules, leghemoglobin, a symbiosis-specific protein, is present (Larrainzar et al. 2020). It binds oxygen and regulates its concentration to ensure optimal nitrogenase activity. This protein also gives the nodule interior a pinkish color, indicating active metabolism (Krishnan et al. 2016; Wang et al. 2023a, b). From an applied perspective, the efficiency of this symbiotic system has been progressively optimized through decades of strain selection and inoculation technologies. Between 1950 and 1955, several Bradyrhizobium strains from various countries were introduced into Rio Grande do Sul, Brazil, to evaluate their nitrogen-fixing efficiency. Following the selection of the most efficient strains, the first private inoculant company was established in the state in 1956 (Freire and Vernetti 1999). Initially, inoculant quality control was regulated by law and overseen by the companies themselves.

Since then, significant advances have been made in agricultural microbial biotechnology, including technologies that improve microbial viability in inoculants (Santos et al. 2019). From 1954 to today, there has been a notable increase in the demand for inoculants containing *Bradyrhizobium* spp. (Freire and Vernetti 1999; Santos et al. 2019), particularly because these bacteria eliminate the need for nitrogen fertilizers (Hungria and Mendes 2015).

Currently, the large-scale adoption of *Bradyrhizobium*-based inoculants reflects their consolidated role in soybean production systems. In 2024, 206 million doses of inoculants were delivered by 20 companies in Brazil, about 57% composed of *Bradyrhizobium* spp., 38% of *Azospirillum* spp., and 5% of *Pseudomonas* spp. (Associação Nacional de Produtores e Importadores de Inoculantes [ANPII] 2025). In the 2022/2023 crop year, soybean was cultivated on 44.1 million hectares (Conab 2025c), with about 85% of this area treated with *Bradyrhizobium* inoculants. Co-inoculation with *Bradyrhizobium* spp. and *Azospirillum* spp. was used on approximately 35% of the soybean area (Associação Paranaense dos Produtores de Sementes e Mudas [APASEM] 2025).

This practice promotes high soybean yields with reduced costs and environmental impact, offering significant economic and ecological benefits (Hungria and Mendes 2015). BNF provides high-value ecosystem services and contributes to more sustainable agriculture when *Bradyrhizobium*-based inoculants are used (Telles et al. 2023). In the 2019–2020 season, BNF saved an estimated BRL 85 billion (~ USD 15 billion) by replacing urea as a nitrogen source (Telles et al. 2023).

Despite these advances, there remains significant potential to further improve BNF efficiency in soybean systems. BNF contributes about 84% of the nitrogen needed by soybean, with the remainder primarily supplied by soil organic matter mineralization. This highlights an opportunity to further enhance BNF efficiency through strategies such as co-inoculation with *Azospirillum* spp. and *Pseudomonas* spp., as well as the use of extracellular membrane vesicles (EMVs). EMVs are promising because they enhance plant–microbe signaling and increase microbial viability in cultivation environments, positioning them as a sustainable alternative to boost BNF’s role in soybean nutrition.

## The role of *Azospirillum* spp. and co-inoculation in sustainable agriculture

The genus *Azospirillum* comprises Plant Growth-Promoting Rhizobacteria (PGPR) that are free-living in the rhizosphere and colonize plant roots without forming nodules (Cassán et al. 2020). These bacteria enhance root development and plant growth through several mechanisms, including the production of phytohormones like auxins, gibberellins, and cytokinins, the biological fixation of atmospheric nitrogen, and the synthesis of siderophores and other signaling compounds (Hungria et al. 2010; Fukami et al. 2016). Additionally, *Azospirillum* spp. can increase the expression of plant genes related to nutrient uptake, stress tolerance, and photosynthesis (Cassán et al. 2020).

One of their most recognized abilities is the synthesis of indole-3-acetic acid (IAA), a phytohormone that promotes the proliferation of lateral roots and root hairs (Lobo et al. 2019; Fukami et al. 2017), enhancing water and nutrient absorption, particularly nitrogen and phosphorus (Cassán et al. 2020; Fukami et al. 2018). Besides IAA, *Azospirillum* spp. produce other bioactive compounds, such as nitric oxide and polyamines, which also stimulate root growth and improve plant resilience to environmental stresses (Vejan et al. 2016). These mechanisms result in increased root surface area and greater nutrient and water uptake efficiency, especially under adverse conditions such as drought or low fertility soils (Fukami et al. 2016; Cassán et al. 2020).

*Azospirillum brasilense* is the most studied and commercially used species within the genus, with strains Ab-V5 and Ab-V6 registered in Brazil and widely adopted in agriculture (Hungria et al. 2010). The adoption of these strains in co-inoculation with *Bradyrhizobium* spp. in soybean crops represents a milestone in tropical agriculture and has shown significant benefits in terms of productivity, resilience, and sustainability (Hungria and Mendes 2015).

Co-inoculation refers to the simultaneous use of *Bradyrhizobium* spp. and *Azospirillum* spp. in seed inoculation or in-furrow application (Hungria et al. 2013). In this system, *Bradyrhizobium* remains the primary symbiont responsible for symbiotic nitrogen fixation, while *Azospirillum* acts as a growth promoter, increasing root development and enhancing the efficiency of BNF and nutrient uptake (Lobo et al. 2019; Fukami et al. 2016). The synergistic interaction between these microorganisms results in higher grain yields, greater root biomass, and improved plant tolerance to abiotic stresses (Fukami et al. 2017; Cassán et al. 2020). Meta-analyses have confirmed yield increases of around 8% in soybean due to co-inoculation, especially under suboptimal water or nutrient conditions (Hungria et al. 2013).

Another important advantage of *Azospirillum* spp. is its adaptability to different soil types and plant species. These bacteria have been successfully used not only in soybean but also in crops such as maize, wheat, rice, and sugarcane (Cassán et al. 2020). Their beneficial effects on plant physiology and development are due not only to direct mechanisms but also to indirect ones, such as the induction of systemic resistance and modulation of the plant microbiome (Vejan et al. 2016; Fukami et al. 2018).

From an ecological perspective, the use of *Azospirillum* spp. contributes to reducing chemical fertilizer use, especially nitrogen fertilizers, which are highly energy-intensive to produce and have significant environmental impacts, such as greenhouse gas emissions and water contamination (Cassán et al. 2020). Therefore, integrating *Azospirillum* spp. and *Bradyrhizobium* spp. into sustainable agricultural systems offers a promising alternative for increasing productivity while reducing environmental risks and production costs.

Despite the recognized benefits of *Azospirillum* spp. and co-inoculation strategies, the field performance of current bioinoculants remains variable and often inconsistent. Limitations such as reduced microbial survival during storage and after seed treatment, sensitivity to environmental stresses (e.g., temperature fluctuations, desiccation, and UV radiation), and incompatibility with chemical seed treatments can compromise their effectiveness. Furthermore, competition with native soil microbiota and limited persistence in the rhizosphere may restrict efficient root colonization, reducing the stability and magnitude of plant growth responses under field conditions. These constraints highlight important technological gaps that need to be addressed to fully exploit the potential of biological inputs in agriculture.

Emerging technologies are enhancing co-inoculation efficiency. One promising innovation is the use of extracellular membrane vesicles (EMVs) derived from beneficial bacteria. These nanostructures act as natural carriers of proteins, lipids, DNA, and signaling molecules (Medina-Castellanos et al. 2023), facilitating plant–microbe communication and improving colonization and activity in the rhizosphere. EMVs can serve as inoculant additives, increasing microbial viability during storage and after field application, improving resilience under stressful conditions, and enabling more stable and efficient biological interactions (Martin et al. 2023). These advances align with the principles of ecological intensification, maximizing the use of biological processes to sustainably increase agricultural productivity (Fukami et al. 2018). In summary, the integration of Azospirillum spp. into sustainable agricultural practices, particularly through co-inoculation with Bradyrhizobium spp., offers a robust strategy for enhancing crop yields, reducing environmental impacts, and promoting biological diversity in production systems. As new technologies like EMVs are developed and adopted, the potential of these microorganisms to revolutionize agriculture becomes even greater, driving a more productive and sustainable future.

## Importance and potential of *Pseudomonas* spp. as a bioinput in soybean

The genus *Pseudomonas* was first described by the pioneer Walter Migula in 1894, marking a significant milestone in microbiology. There are 452 distinct species described within this genus (Fan et al. 2025). These bacteria stand out for their remarkable ability to adapt to various environmental stresses (Khoshru et al. 2025). Species of the genus *Pseudomonas* are widely recognized for their plant growth-promoting activities. Species such as *P. syringae*, *P. putida*, *P. chlororaphis*, *P. protegens*, and *P. fluorescens* have been used in agriculture with significant roles in controlling phytopathogens through the secretion of various secondary metabolites, hormones, and enzymes (Khoshru et al. 2025). *Pseudomonas* spp. bacteria have been extensively researched, especially for their ability to produce antimicrobial metabolites, as well as their potential to suppress phytopathogens, stimulate plant growth, produce enzymes and hormones, and solubilize inorganic minerals (Shahid et al. 2018). These microorganisms are widely used in agriculture as plant growth-promoting bacteria (PGPB), playing a crucial role in controlling plant diseases (Zhang et al. 2023).

Some species stand out due to multiple modes of action related to the biocontrol of fungal phytopathogens in soybean, mainly through genes associated with antifungal activity (León et al. 2009). Others demonstrate high potential in saline soil environments, being effective in root colonization and consequently promoting seed germination, seedling vigor, and aerial growth under adverse conditions (Costa-Gutierrez et al. 2021). For example, *P. simiae* is capable of alleviating water stress by protecting plants through modulation of gene expression and production of hormones that confer greater drought tolerance (Vaishnav and Choudhary 2019). Species like *P. koreensis* promote the solubilization of silicate and phosphate even in saline environments, modulating soybean internal physiology (Adhikari et al. 2020).

Overall, *Pseudomonas* spp. positively influence various morphological and physiological aspects of soybean, including stem and root elongation, increased fresh and dry plant biomass, as well as biochemical parameters (Dubey et al. 2021). These effects result in higher productivity, improved microbial community in the rhizosphere and root system, and enhanced soil characteristics (Zhang et al. 2023). Specifically, *P. putida*, when colonizing soybean roots, promotes aerial growth, positively influencing chlorophyll synthesis and plant hormones (Teiba et al. 2023; Costa-Gutierrez et al. 2020). This microorganism also favors growth under salt stress by producing hormones, osmoprotectants, and activating antioxidant enzymes (Teiba et al. 2023). These effects benefit root architecture, improve water and nutrient absorption, and support aerial development (Costa-Gutierrez et al. 2020). With the expansion of soybean cultivation areas, the use of microorganisms like *Pseudomonas* spp. represents a highly sustainable strategy with great potential to improve crop productivity and resilience (Egamberdieva et al. 2017).

*Bradyrhizobium* spp. bacteria are responsible for symbiotic nitrogen fixation in soybean (Hungria and Mendes 2015), while *Azospirillum* spp. enhance root growth, expanding the plant's ability to absorb water and nutrients (Cassán et al. 2020). *Pseudomonas* spp. play an important role in phosphorus solubilization and production of plant hormones that stimulate growth (Egamberdieva et al. 2017). Coinoculation of these three bacteria is an efficient alternative, as it increases the number of nodules due to more infection points provided by the expanded root system, resulting in greater water and nutrient uptake as well as phosphorus solubilization, which contributes to increased phosphorus and nitrogen content in the plant. These benefits can be further enhanced with the use of extracellular membrane vesicles (EMVs).

## Coinoculation of *Bradyrhizobium*, *Azospirillum*, and *Pseudomonas*

The practice of coinoculation in soybean cultivation boosts agricultural efficiency, providing economic and environmental advantages while substantially increasing productivity (Barbosa et al. 2021; Deak et al. 2022). Beyond these agronomic outcomes, the effectiveness of coinoculation is fundamentally driven by synergistic and complementary interactions between microorganisms that operate at molecular, physiological, and ecological levels. These interactions involve coordinated modulation of plant signaling pathways, enhancement of rhizosphere competence, and optimization of resource-use efficiency. In this context, coinoculation does not simply represent the additive effect of two microorganisms, but rather a systems-level interaction in which microbial consortia reshape plant physiology and rhizosphere functionality (Backer et al. 2018). In the future, inoculation or coinoculation using extracellular membrane vesicles (EMVs) combined with PGPB may further potentiate these effects, acting as carriers of signaling molecules, enzymes, and nucleic acids that enhance plant–microbe communication and microbial establishment (Rutter and Innes 2017). This integrated approach reinforces coinoculation as a strategic tool to optimize soybean production with increased sustainability. Its adoption has expanded significantly in Brazil, resulting in consistent improvements in plant morphology, root architecture, and yield stability (Moretti et al. 2020a).

Plant growth-promoting bacteria (PGPB) are widely recognized for their ability to modulate plant development through multiple biochemical pathways. For instance, Pseudomonas putida KT2440 (strains mus-20, mus-42, and EU206) can solubilize inorganic phosphate through the secretion of organic acids, synthesize siderophores that enhance iron acquisition, and produce indole-3-acetic acid (IAA), a key auxin regulating cell elongation and root development (Costa-Gutierrez et al. 2020; Miransari and Smith 2014). When coinoculated with *Bradyrhizobium japonicum* USDA110, these traits lead to synergistic improvements in plant growth, including increases in root length, shoot biomass, nodulation, and phosphorus uptake (Egamberdieva et al. 2017). Mechanistically, these responses are associated with enhanced rhizosphere colonization, increased root exudation, and improved nutrient fluxes, which collectively create a more favorable microenvironment for symbiotic establishment.

Bacteria of the genus *Azospirillum* are among the most widely used PGPB globally, although important gaps remain regarding their molecular interaction with rhizobia during coinoculation (Hungria et al. 2015). Evidence indicates that coinoculation promotes significant increases in nodule number and biomass, largely due to early modulation of root system architecture. Coinoculation of B. japonicum with *A. brasilense* stimulates lateral root formation and root hair proliferation, increasing the number of infection sites available for rhizobial entry and accelerating the onset of nodulation (Chibeba et al. 2015; Vurukonda et al. 2016). This effect is closely linked to auxin-mediated signaling pathways and alterations in root exudate composition.

At the molecular level, coinoculation with *Azospirillum* spp. enhances the production of phytohormones such as indole-3-acetic acid (IAA), which promotes root elongation and root hair differentiation, thereby increasing infection points. Simultaneously, these changes stimulate the secretion of flavonoids by plant roots, which act as key signaling molecules inducing the expression of rhizobial nodulation (nod) genes (Queiroz Rego et al. 2018; Rondina et al. 2020). This coordinated signaling triggers a cascade of events including Nod factor perception by plant receptors, calcium spiking, activation of symbiotic gene networks, cortical cell division, infection thread formation, and ultimately nodule organogenesis (Masson-Boivin et al. 2009a, b). Thus, coinoculation enhances both the efficiency and timing of symbiotic establishment, leading to increased biological nitrogen fixation (BNF).

In addition to early signaling events, coinoculation influences plant hormonal homeostasis at a systemic level. The combined presence of *Azospirillum* spp. and *Bradyrhizobium* spp. can alter endogenous concentrations of auxins, cytokinins, and gibberellins, resulting in more balanced growth regulation compared to single inoculation (Cassán et al. 2009; Cassán and Diaz-Zorita 2016). This hormonal crosstalk is critical not only for root development but also for coordinating shoot growth and source–sink relationships. Furthermore, coinoculation has been associated with the modulation of ethylene levels through ACC deaminase activity, reducing stress-induced inhibition of root growth and improving plant resilience under abiotic stress conditions (Glick 2014).

Coinoculation also enhances plant tolerance to environmental stresses by activating antioxidant defense systems and regulating stress-responsive gene expression. Increased activity of enzymes such as superoxide dismutase, catalase, and peroxidases contributes to the mitigation of oxidative damage under conditions such as drought and salinity (Chieb and Gachomo 2023). In parallel, improvements in relative water content and membrane stability have been observed, indicating enhanced cellular integrity and physiological performance under water deficit (Silva et al. 2019). These responses highlight the role of coinoculation in improving plant stress physiology through integrated biochemical and molecular mechanisms.

From a nutritional perspective, coinoculation significantly enhances nitrogen accumulation in plant tissues due to improved BNF efficiency and greater nodulation (Cerezini et al. 2016). *Bradyrhizobium* spp. directly contributes to nitrogen fixation, while Azospirillum spp. indirectly supports this process by improving root system architecture and increasing nutrient and water uptake (Hungria et al. 2015; Cerezini et al. 2016). This complementary interaction results in increased root length, volume, and surface area, as well as higher nodule number and mass, ultimately leading to greater grain productivity compared to single inoculation (Rondina et al. 2020).

In addition, coinoculation promotes the production of secondary metabolites that are associated with improved plant performance and grain quality. These include phenolic compounds, flavonoids, and other metabolites involved in plant defense and signaling (Moretti et al. 2020b). The enhanced supply of nitrogen via BNF plays a central role in protein synthesis, directly influencing grain protein content and yield (Oliveira et al. 2020; Acuña et al. 2020). Field studies have demonstrated that coinoculation can increase soybean productivity by up to 31% compared to non-inoculated treatments (Fipke et al. 2020), highlighting its agronomic relevance.

At the ecological level, coinoculation can reshape the rhizosphere microbiome by promoting beneficial microbial interactions and suppressing pathogenic organisms. This restructuring is driven by changes in root exudation patterns, microbial competition, and niche differentiation, resulting in a more stable and resilient soil microbial community (Trivedi et al. 2020). Such microbiome-level effects further contribute to long-term soil health and sustainability.

Despite its well-documented benefits, coinoculation still faces challenges related to formulation stability, compatibility between microbial strains, and variability in field performance under different environmental conditions. Achieving consistent results requires a deeper understanding of microbial interactions, signaling pathways, and environmental influences on symbiosis. Nevertheless, coinoculation represents a promising and sustainable strategy for enhancing agricultural productivity while reducing dependence on chemical fertilizers. Its ability to integrate biological nitrogen fixation, plant growth stimulation, and stress tolerance mechanisms positions it as a key component of future sustainable agricultural systems.

## Molecular vesicles and their bacterial-derived compounds

**Fig. 1:**
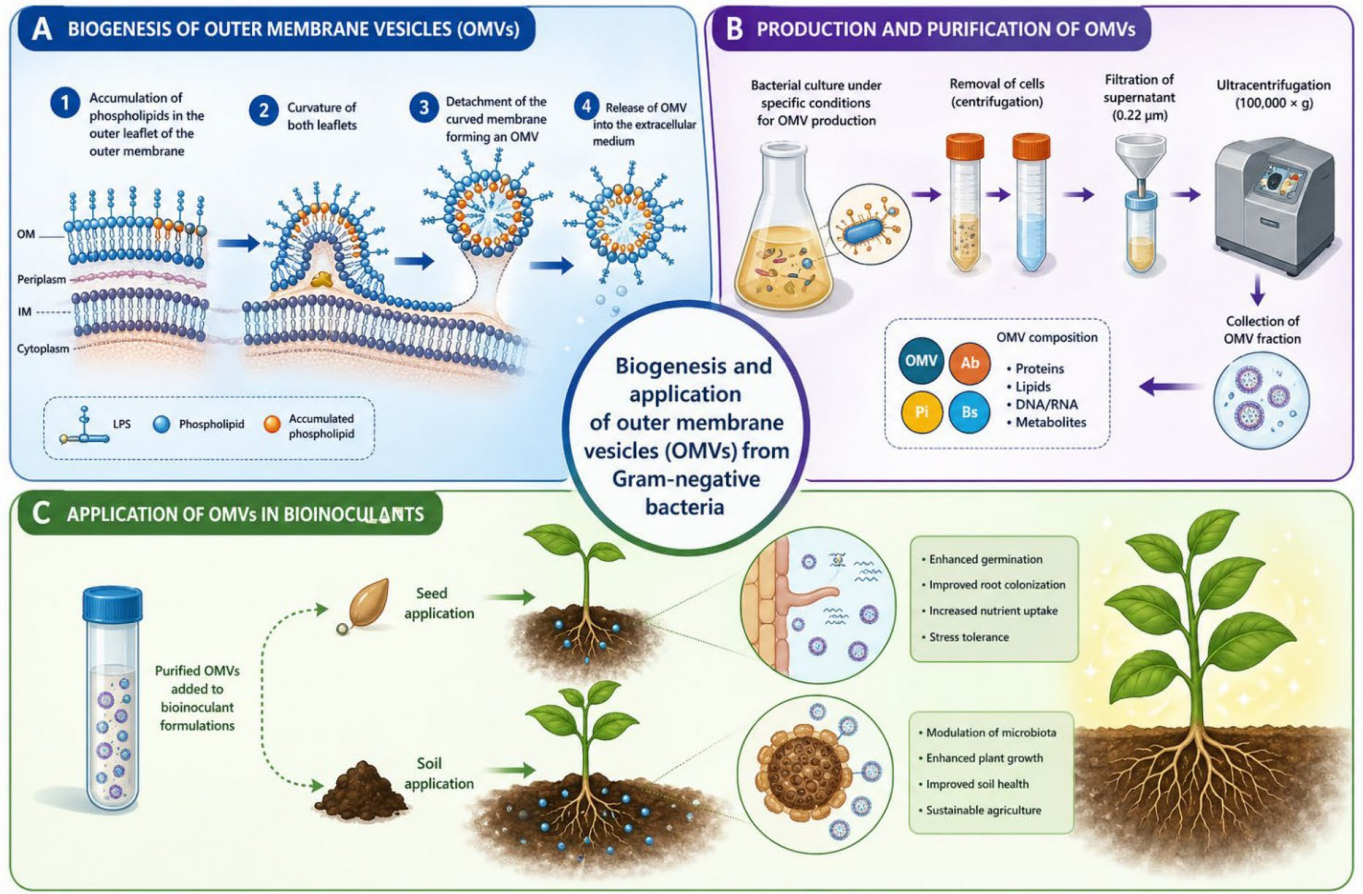
Biogenesis of outer membrane vesicles in gram-negative bacteria. In the process, there is (1) accumulation of phospholipids in the outer leaflet of the outer membrane, causing a disturbance in the outer membrane structure. The continuous enrichment of phospholipids in the outer membrane leads to (2) curvature of both leaflets, which then (3) detach from the cell, forming a outer membrane vesicle (OMV), and then (4) release of OMV into the extracellular medium (**A**); growth of gram-negative bacteria under specific conditions to produce outer membrane vesicles, which go from the process of removing the cells and filtering the supernatants to remove any remaining cells, to ultracentrifugation to collect vesicle fractions (**B**). After extraction of the outer membrane vesicles, they will be added to bioinoculants that can be applied via seeds or in the soil (**C**). Modified from Borrero de Acuña and Bernal (2021).

Extracellular membrane vesicles (EMVs), also referred to as extracellular vesicles (EVs), are nanoscale, spherical structures typically ranging from 20 to 400 nm in diameter, delimited by a lipid bilayer and released by organisms across all domains of life (Fig. 1) (Jiménez‐Guerrero et al. 2023; Toyofuku et al. 2023; Gill et al. 2019). In bacteria, particularly Gram-negative species, EMVs originate mainly from two distinct biogenesis pathways: controlled blebbing of the outer membrane and explosive cell lysis, both of which result in the release of membrane-bound vesicles containing selectively enriched molecular cargo (Toyofuku et al. 2019; Turnbull et al. 2016). Due to their unique composition and mode of formation, EMVs are increasingly recognized as a specialized and evolutionarily conserved secretion pathway, often referred to as the type 0 secretion system (T0SS), distinct from classical secretion systems (Guerrero-Mandujano et al. 2017).

Structurally, EMVs encapsulate a diverse array of biomolecules, including proteins, lipids, nucleic acids (DNA and RNA), signaling compounds, enzymes, and secondary metabolites. Importantly, the vesicular cargo is not a random subset of the cytoplasm but rather a selectively packaged molecular repertoire, often enriched in bioactive compounds involved in intercellular communication and environmental adaptation (Biller et al. 2022; Villageliu and Samuelson 2022). This selective packaging enables EMVs to function as highly efficient delivery systems, ensuring protection of labile molecules from extracellular degradation, maintenance of effective concentrations, and coordinated delivery of multiple compounds to target cells, a process described as “quantal secretion” (Toyofuku et al. 2023; Bitto et al. 2017; Renelli et al. 2004; Wang et al. 2023a, b).

In the context of plant–microbe interactions, EMVs represent a sophisticated mechanism of molecular exchange and signaling. These vesicles can be internalized by plant cells or interact with cell surface receptors, thereby modulating host physiological and molecular responses (Cai et al. 2023; Guerrero-Mandujano et al. 2017; Jiménez-Guerrero et al. 2023). Their roles extend to multiple biological processes, including delivery of effector molecules, horizontal gene transfer, antimicrobial activity, and immunomodulation (Fulsundar et al. 2014; Kadurugamuwa and Beveridge 1996; Schertzer and Whiteley 2012). This multifunctionality positions EMVs as central mediators of both beneficial and pathogenic plant–bacteria interactions.

In symbiotic systems such as the rhizobium–legume interaction, EMVs are emerging as an additional regulatory layer in the molecular dialogue that governs nodulation. Classical symbiosis is initiated by the release of flavonoids from plant roots, which activate rhizobial nodulation genes via the NodD regulator, leading to the synthesis of Nod factors (NFs)—lipo-chitooligosaccharides that trigger nodule organogenesis (Masson-Boivin et al. 2009a, b; Fisher and Long 1989; Spaink et al. 1995; Zipfel and Oldroyd 2017). Recent evidence suggests that EMVs may act as carriers of these signaling molecules, facilitating their delivery in a protected and concentrated form. For example, vesicles produced by Rhizobium etli and Sinorhizobium fredii under flavonoid induction have been shown to induce root hair deformation, indicating the presence of biologically active NFs within EMVs (Taboada et al. 2019; Li et al. 2022). This suggests that vesicle-mediated transport may enhance signaling efficiency and spatial targeting during early symbiotic events.

Beyond Nod factors, EMVs produced by rhizobia can transport additional symbiosis-related molecules, including surface polysaccharides, enzymes, and regulatory RNAs, all of which contribute to successful infection thread formation and nodule development (Jiménez-Guerrero et al. 2022; Salinero-Lanzarote et al. 2019; Reyes-Pérez et al. 2025). Rhizobial EMVs have also been shown to encapsulate small RNAs (sRNAs), which can be transferred to plant cells and potentially modulate host gene expression, representing a novel mechanism of cross-kingdom regulation (Ayala-García et al. 2024b). However, the precise role of EMVs during different stages of symbiotic infection remains only partially understood and represents a key frontier in plant–microbe interaction research.

In addition to their role in symbiosis, EMVs are involved in modulating plant immunity. Vesicles can carry microbe-associated molecular patterns (MAMPs), enzymes, and signaling compounds capable of triggering plant defense responses or, alternatively, promoting immune tolerance depending on the context (Pandey et al. 2024; Bahar et al. 2016). This dual role highlights their importance in balancing defense and symbiosis. Furthermore, proteins and metabolites transported within EMVs are shielded from extracellular proteases and environmental degradation, enhancing their stability and persistence in the rhizosphere.

Experimental studies have demonstrated that EMVs derived from plant growth-promoting bacteria (PGPB) can exert systemic effects on plant physiology. For instance, vesicles from *Azospirillum* sp. B510 have been shown to induce the expression of defense-related genes and stimulate the production of antimicrobial metabolites, thereby enhancing resistance against pathogens (Zannis-Peyrot et al. 2025). Similarly, EMVs from *Pseudomonas fluorescens* contain proteins associated with stress tolerance, metabolic adaptation, and plant interaction, contributing to both plant growth promotion and microbial fitness (Mcmillan and Kuehn 2023).

From a biotechnological perspective, EMVs represent a promising tool for next-generation inoculant development. Their ability to deliver concentrated and protected bioactive compounds opens new possibilities for designing bioformulations that enhance plant growth, nodulation, and stress tolerance. The production process involves bacterial cultivation under controlled conditions, followed by vesicle isolation through filtration and ultracentrifugation, and subsequent characterization in terms of structure, concentration, and biological activity (Zannis-Peyrot et al. 2025; Bitto et al. 2021; Jiménez‐Guerrero et al. 2023). Importantly, vesicle production is influenced by bacterial species, growth conditions, and environmental factors, which must be optimized to ensure reproducibility and efficacy.

Despite their significant potential, the application of EMVs in agriculture remains at an early stage. While in vitro and controlled environment studies demonstrate their capacity to enhance plant growth and symbiotic efficiency, there is still a lack of field-based evidence, particularly in major crops such as soybean. Future research should focus on understanding dose–response relationships, interaction with native microbiomes, and integration with coinoculation strategies involving beneficial microorganisms such as *Bradyrhizobium* and *Azospirillum*. In this context, EMVs may act as molecular amplifiers of plant–microbe interactions, representing a novel and promising frontier for sustainable agricultural intensification.

## Practical applications and future challenges

In the context of the growing global demand for food, driven by rapid population expansion (Cao and Wang 2025), modern agriculture faces the challenge of increasing productivity while reducing environmental impacts. Historically, agricultural intensification has relied heavily on synthetic fertilizers and pesticides to ensure plant nutrition and crop protection (Timsina 2018). However, the continuous and excessive use of these inputs has raised significant environmental concerns, particularly regarding the contamination of soil, water, and air resources (Timsina 2018). In this scenario, the transition toward more sustainable production systems is no longer optional but essential, requiring the adoption of innovative strategies that balance productivity with environmental conservation (Chojnacka et al. 2020).

Among these strategies, the use of biofertilizers based on beneficial microorganisms has emerged as a key component of sustainable agriculture (Mącik et al. 2020). These biological inputs contribute not only to improved nutrient use efficiency but also to enhanced plant resilience under biotic and abiotic stresses, including those associated with climate change (Bessai et al. 2021; Soares et al. 2023). Microbial inoculants can stimulate plant growth through multiple mechanisms, including antibiosis against pathogens, production of phytohormones, enzymes, and secondary metabolites, as well as improvement of soil fertility and nutrient cycling processes (Ayilara et al. 2023; Mącik et al. 2020; Soares et al. 2023).

Within this context, inoculants formulated with plant growth-promoting bacteria (PGPB) have become widely adopted, particularly in legume crops such as soybean (Bessai et al. 2021; Soares et al. 2023). In Brazil, these products are defined as formulations containing microorganisms capable of promoting plant growth (Brazil 2010), with a strong predominance of rhizobial inoculants that establish symbiotic relationships with Fabaceae species (Bomfim et al. 2021; Mącik et al. 2020). This well-established interaction, recognized for over a century, results in root nodule formation and efficient biological nitrogen fixation (BNF), enabling partial or even complete replacement of nitrogen fertilizers in crops such as soybean (Hungria and Mendes 2015; Kebede 2021). The large-scale adoption of these technologies is evidenced by the commercialization of more than 70 million doses of *Bradyrhizobium*-based inoculants in a single growing season, with a continuous annual market growth estimated at 16% (Santos et al. 2019; Telles et al. 2023).

Despite these advances, the consolidation of inoculants as a central pillar of sustainable agriculture still depends on overcoming several technical, economic, and regulatory challenges. These include ensuring product quality and stability, improving formulation technologies, optimizing application methods under diverse environmental conditions, and reducing production costs associated with research and innovation (Díaz-Rodríguez et al. 2025; Hossain et al. 2023). In addition, regulatory frameworks, such as those established in Brazil, play a crucial role in standardizing product quality but may also represent barriers due to their complexity and associated costs (Brazil 2011; Dos Reis et al. 2024).

In this evolving scenario, new technological approaches are emerging and reshaping the future of inoculant development. Molecular inoculants, for example, incorporate bioactive molecules or modified microbial components, aiming to enhance bacterial performance, root development, nodulation efficiency, and plant immunity (Bitto et al. 2021). Although still at an early stage, these innovations point toward a new generation of bioinputs capable of integrating advanced biological functions into agricultural systems. Similarly, the use of microbial consortia has gained prominence, based on the combination of microorganisms with complementary functional traits, enabling more robust and adaptable responses under variable environmental conditions (Liu et al. 2023; Nunes et al. 2024; Timofeeva et al. 2023).

Advances in genetic improvement technologies further reinforce this perspective. Genome editing tools such as CRISPR enable precise manipulation of microbial genomes, allowing the development of strains with enhanced stress tolerance, improved symbiotic efficiency, and greater adaptability to environmental constraints (Kursheed et al. 2024; Serantes et al. 2024). This approach opens new possibilities for the development of “climate-smart” inoculants, capable of maintaining high performance under increasingly challenging agricultural conditions.

Thus, although the use of microbial inoculants is already well established in crops such as soybean, their future development lies in the integration of advanced biotechnologies, improved formulations, and a deeper understanding of plant–microbe interactions. These advances are expected to consolidate inoculation and coinoculation strategies as essential tools for sustainable intensification, contributing not only to increased productivity but also to the resilience and long-term sustainability of agricultural systems.

## Conclusion

The use of inoculants containing plant growth-promoting bacteria (PGPB) represents an effective strategy to mitigate the negative effects associated with the intensive use of chemical fertilizers, climate change, diseases and pests, as well as soil acidification, thus promoting progress toward more sustainable agriculture. Beneficial PGPB, when introduced into the soil, positively modulate the functioning of the rhizosphere.

The characterization and selection of these bacteria are complex processes, but recent technological advances have facilitated this challenge, helping researchers identify bacterial communities and assess their functional potential. It is essential to understand the mechanisms involved in these bacteria’s capacity to promote plant growth, especially in soybean cultivation.

Deepening knowledge about PGPB and their functions, as well as understanding the physiological and molecular mechanisms they trigger, will enable more efficient use of these microorganisms. Moreover, their use in biological control of pests and diseases, as well as partial replacement of chemical fertilization, becomes possible, reducing dependence on synthetic inputs.

In light of this innovation trend, there is an increasing number of available technologies, including the development of commercial inoculant products, both in the form of microbial consortia and through the application of genetic editing tools. In the future, the use of inoculants based on bacterial vesicles may establish itself as a promising alternative for agriculture.

Although there are currently various inoculant technologies available, there remains a constant need to pursue new knowledge, driven by intense pressure from new discoveries and growing understanding of the mechanisms involved. This continuous process is essential to achieve increasingly sustainable agricultural production systems.

## Data Availability

The document used to support the review of this paper is available from the corresponding author upon reasonable request.
